# Sarcoidosis and Multiple Myeloma: A Case Report and Literature Review

**DOI:** 10.1155/2019/4586265

**Published:** 2019-10-30

**Authors:** Joao Tiago Serra, Aurelia Martinho, Fernanda Paixao Duarte, Fernando Aldomiro

**Affiliations:** Department of Internal Medicine II, Professor Doutor Fernando Fonseca Hospital, Amadora, Portugal

## Abstract

The existence of a sarcoidosis-lymphoma syndrome has been previously proposed since the relation between sarcoidosis and an increased risk of lymphoproliferative disorders is well established. Multiple myeloma is a malignant multifocal proliferation of clonal plasma cells within the bone marrow, and its association with sarcoidosis has been rarely described. We present a concurrent diagnosis of sarcoidosis and multiple myeloma and make a brief analysis of the reported cases in the literature. A 65-year-old woman underwent surgery for the excision of a wrist mass that presented 3 years before. Histological analysis showed sarcoid-type epithelioid granulomas without necrosis, establishing soft tissue sarcoidosis. Further evaluation revealed marked interstitial lung parenchyma lesions and large intrathoracic adenopathies. Bronchofibroscopy with transbronchial biopsy confirmed lung sarcoidosis. In addition, blood analysis showed monoclonal IgG kappa gammopathy. A bone marrow biopsy confirmed hypercellularity with 60% plasma cells and plasmocyte infiltration. Thus, the diagnosis of systemic sarcoidosis and multiple myeloma was established simultaneously. In a brief review of the literature, we identified 33 reports of cases with both sarcoidosis and multiple myeloma. We point out the importance of a high level of suspicion for the association of sarcoidosis with malignant haematological diseases such as multiple myeloma.

## 1. Introduction

Sarcoidosis is a T-cell-mediated immunological response against an unknown environmental trigger in a susceptible host. Noncaseating granulomas, consisting of compact and centrally organized collections of CD4+ T cells, macrophages, and epithelioid cells, are the hallmark of sarcoidosis and the outcome of an unbalanced T-helper 1 immune response [1, 2]. Multiple myeloma (MM) is a malignant multifocal proliferation of clonal plasma cells within the bone marrow, associated with skeletal destruction, serum monoclonal gammopathy, and end-organ sequelae [3]. Brincker [4] described the sarcoidosis-lymphoma syndrome in 1986 for the particular association between sarcoidosis and lymphoproliferative disorders (LD); however, the association between sarcoidosis and MM has been rarely reported and the explanation for this relation remains unclear and controversial. We describe a case of sarcoidosis associated with MM and present a brief analysis of published cases in the literature.

## 2. Case Report

A 65-year-old Caucasian women, retired cook, was referred to our outpatient clinic due to a three-year history of a painless mass on the dorsal side of the right wrist. Her medical background was positive for type 2 diabetes mellitus, dyslipidaemia, bronchitis, and venous thrombosis of the right eye. The wrist lesion had been growing gradually and affected finger movement. There was no history of blunt or penetrating trauma.

**Figure 1:**
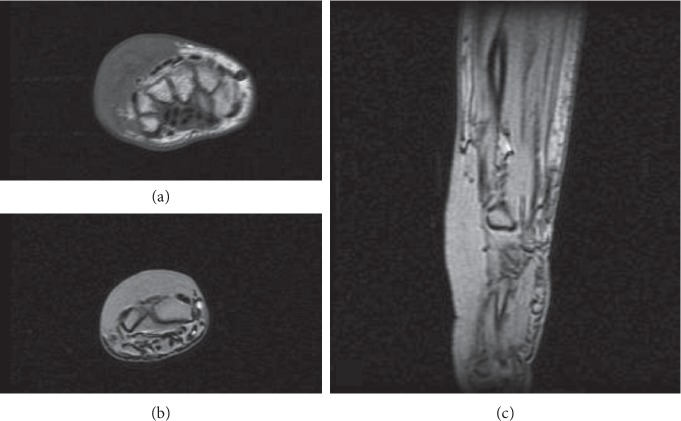
Magnetic resonance images in T1-weighted axial view (a), T2-weighted axial view (b), and T2-weighted sagital view (c), identifying a lesion on the dorsal plane of the right wrist, extending to the inner face but without vascular or tendinous invasion, measuring about 100 × 60 × 18 mm in diameter, with well-defined limits.

An ultrasound, complemented with nuclear magnetic resonance, identified a lesion on the dorsal plane of the right wrist, extending to the inner face, but without vascular or tendinous invasion, measuring about 100 × 60 × 18 mm in diameter and having well-defined limits (Figure 1). After surgery for lesion removal, histological analysis showed an extensive granulomatous process without necrosis, consisting of sarcoid-type epithelioid granulomas. The patient also complained of long-term intermittent nodular skin lesions on both legs, dry cough, and dyspnoea, for which she had been previously prescribed bronchodilators with little symptomatic relief. Physical examination revealed painless bilateral supraclavicular lymphadenopathies, bibasilar coarse crackles, and nodular skin lesions scattered along both inferior limbs (*erythema nodosum*). There were no other palpable lymph nodes, hepatosplenomegaly, fever, night sweats, or constitutional symptoms.

Laboratory studies showed haemoglobin 9.7 g/dL (reference range 11.5–16.5 g/dL), white blood cells count of 4.6 × 10^9^/L (reference range 4–11 × 10^9^/L), C-reactive protein of 0.87 mg/dL (reference range <0.30 mg/dL), erythrocyte sedimentation rate of 83 mm (reference range <20 mm), serum angiotensin converting enzyme (ACE) of 141.28 U/L (reference range 12–68 U/L), albumin of 3.66 g/dL (reference range 3.97–4.94 g/dL), and total serum protein level of 8.40 g/dL (reference range 6.40–8.20 g/dL). Serum protein electrophoresis revealed a monoclonal band, confirmed by immunofixation to be immunoglobulin IgG kappa. Further quantification of serum immunoglobulins showed an elevated IgG level of 3120 mg/dL (reference range 70–1600 mg/dL), with normal IgM and IgA, and beta-2 microglobulin of 2.09 mg/dL (reference range 0.67–1.31 mg/dL). Mantoux assay and Bence Jones proteinuria quantification were negative, and both renal function and serum calcium were within the normal range. The screening for HIV and hepatitis B and C was negative.

**Figure 2:**
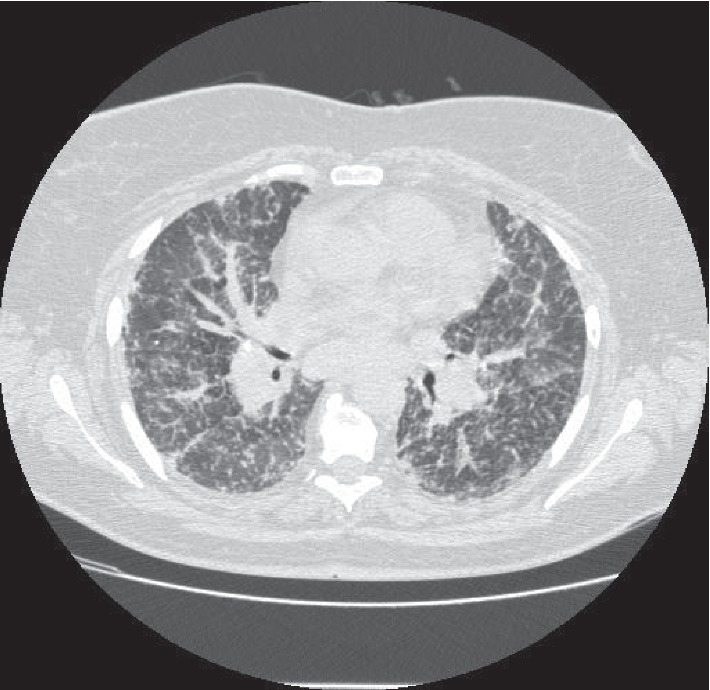
Chest high-resolution computed tomography image showing marked and diffuse alteration of pulmonary parenchyma permeability, thickening of the interlobular septa, and fine bronchovascular and subpleural micronodularity, associated with mediastinal lymphadenopathy.

A high-resolution computed tomography of the chest showed large mediastinal and axillary adenopathies with extensive conglomerates, associated with marked and diffuse permeability of the entire pulmonary parenchyma, thickening of the interlobular septa, and fine bronchovascular and subpleural micronodularity (Figure 2).

**Figure 3:**
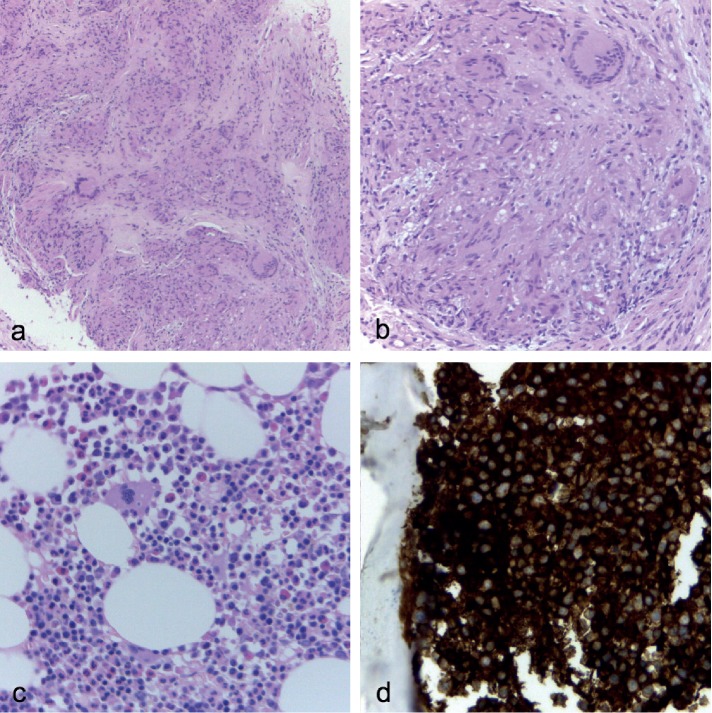
Histological analysis: transbronchial biopsy (a, b) revealing granulomatous inflammatory process compatible with lung sarcoidosis; bone marrow biopsy showing hypercellularity with plasmocyte infiltration (c) and kappa light chain restriction (d).

The patient's lung function testing revealed a mild restrictive pattern with decreased diffusion capacity. A bronchofibroscopy with transbronchial biopsy and bronchioalveolar lavage was performed. Respiratory tract fluid analysis showed an increased CD4/CD8 ratio of 9.02, and both acid-fast staining (Ziehl–Neelsen coloration) and mycobacterial culture were negative. Bronchial biopsy histological results revealed mild chronic inflammatory infiltrate with multiple nonnecrotizing sarcoid-type epithelioid granulomas and giant multinucleated Langerhans cells (Figures 3(a) and 3(b)). The patient also underwent a scintigraphy with gallium 67, which documented pathological uptake in the lungs, axillary and inguinal regions, and several cutaneous foci on the lower limbs. Ophthalmologic evaluation was consistent with ocular sarcoidosis. For further evaluation of serum monoclonal IgG kappa gammopathy, a bone marrow aspirate and biopsy were performed, showing a hypercellular bone marrow with 60% plasma cells and plasmocyte infiltration with K light chain restriction, respectively (Figures 3(c) and 3(d)).

Hence, a simultaneous diagnostic of systemic sarcoidosis and multiple myeloma stage I according to the International Staging System was established. The patient was started on prednisolone 60 mg/day with clinical and biochemical improvement. Bone marrow plasma cell infiltration dropped to 28%, and IgG immunoglobulin and ACE returned to normal values. Although a slow reduction of steroid therapy (to 5 mg/day) was achieved in time, previous oral antidiabetic therapy became insufficient and prompted insulin introduction.

## 3. Discussion

Only few cases of sarcoidosis in association with MM have been reported in the literature, so there is not enough scientific evidence available to support a causal relation for the occurrence of these two diseases. Nonetheless, epidemiological data highlight the higher risk for lymphoproliferative disease development in sarcoidosis patients [5–8].

Brincker and Wilbek [5] published the first study regarding the association between sarcoidosis and malignant diseases in 1974. In a retrospective analysis of 2544 Danish sarcoidosis patients, the risk of lymphoproliferative diseases was found to be 11.5 times higher than expected, the most common being Hodgkin's disease. That same year, Selroos et al. [9] reported the first known case of sarcoidosis associated with MM, in a 56-year-old Finnish woman. Based on previous publications, Brincker [4] hypothesised the existence of a sarcoidosis-lymphoma syndrome, anchored on the following features: lymphoproliferative disorders always followed sarcoidosis, the median age of sarcoidosis diagnosis in patients with lymphoma was 10 years later than those with sarcoidosis diagnosed first, and B-cell Hodgkin's malignancy was more frequent then T-cell lymphomas.

In our review of the literature, 33 cases with both sarcoidosis and MM were identified although patient characteristics and diagnostic information were not available for all of them [4, 6–25]. Data analyses showed female gender predominance, with a median age at sarcoidosis diagnosis of 46 years (24–65). Despite being about 10 years lower than previous authors have reported, it is still higher than the median age for sarcoidosis diagnosis in the general population [3, 16, 19]. The median time interval between sarcoidosis and MM diagnosis was 8.2 years (0–27), but in 9 cases a simultaneous diagnosis of both diseases was reported. These data are in favour of Brincker's [4] hypothesis that malignant haematological diseases are associated with chronic active sarcoidosis in middle-aged patients. Also in accordance with this predisposing relation is the fact that in almost all patients, sarcoidosis preceded the diagnosis of MM. With respect to MM, IgG was the most common monoclonal protein reported, followed by IgA and IgM.

The explanation for the relationship between sarcoidosis and lymphoproliferative disorders, particularly MM, is still controversial and unknown. One possible link between sarcoidosis and MM is the polyclonal hypergammaglobulinemia induced by sarcoidosis chronic inflammatory response. This continuous state of stimulation results in prolonged B cell and plasma cell half-life, which in addition to immune system imbalance in sarcoidosis patients, predisposes for genetic mutations and might lead to haematological malignancy development [16, 20]. Recent studies have highlighted the potential role of T_H_17 cells and T_reg_ cells in sarcoidosis and MM immune mechanisms [1]. Despite MM being a primary B-cell malignancy, both quantitative and functional T-cell abnormalities have been demonstrated to occur in this disease.

In our case, sarcoidosis diagnosis was first made after a soft tissue lesion excision in a 65-year-old woman. Although both sarcoidosis and MM were diagnosed simultaneously, previous respiratory symptoms suggest that sarcoidosis pulmonary involvement was already present years before and misdiagnosed as bronchitis. This fact might also account for the older age at the time of the diagnosis of sarcoidosis. No usual abnormalities related to MM, such as lytic lesions or renal function impairment, were present at the time of diagnosis, and only the serum protein electrophoresis monoclonal band called the attention to a possible underlying disease other than sarcoidosis. We aim to call the attention to the possibility of LD development in patients with sarcoidosis. A high level of suspicion should always be present both at the time of diagnosis as well as during follow-up since LD seems to develop years after the diagnosis of sarcoidosis.
